# Assessing when chromosomal rearrangements affect the dynamics of speciation: implications from computer simulations

**DOI:** 10.3389/fgene.2014.00295

**Published:** 2014-08-26

**Authors:** Jeffrey L. Feder, Patrik Nosil, Samuel M. Flaxman

**Affiliations:** ^1^Department of Biological Sciences, University of Notre DameNotre Dame, IN, USA; ^2^Department of Animal and Plant Sciences, University of SheffieldSheffield, UK; ^3^Department of Ecology and Evolutionary Biology, University of ColoradoBoulder, CO, USA

**Keywords:** genomic architecture, inversions, linkage disequilibrium, genetic hitchhiking, speciation-with-gene-flow, genomic islands of divergence, ecological speciation, models

## Abstract

Many hypotheses have been put forth to explain the origin and spread of inversions, and their significance for speciation. Several recent genic models have proposed that inversions promote speciation with gene flow due to the adaptive significance of the genes contained within them and because of the effects inversions have on suppressing recombination. However, the consequences of inversions for the dynamics of genome wide divergence across the speciation continuum remain unclear, an issue we examine here. We review a framework for the genomics of speciation involving the congealing of the genome into alternate adaptive states representing species (“genome wide congealing”). We then place inversions in this context as examples of how genetic hitchhiking can potentially hasten genome wide congealing. Specifically, we use simulation models to (i) examine the conditions under which inversions may speed genome congealing and (ii) quantify predicted magnitudes of these effects. Effects of inversions on promoting speciation were most common and pronounced when inversions were initially fixed between populations before secondary contact and adaptation involved many genes with small fitness effects. Further work is required on the role of underdominance and epistasis between a few loci of major effect within inversions. The results highlight five important aspects of the roles of inversions in speciation: (i) the geographic context of the origins and spread of inversions, (ii) the conditions under which inversions can facilitate divergence, (iii) the magnitude of that facilitation, (iv) the extent to which the buildup of divergence is likely to be biased within vs. outside of inversions, and (v) the dynamics of the appearance and disappearance of exceptional divergence within inversions. We conclude by discussing the empirical challenges in showing that inversions play a central role in facilitating speciation with gene flow.

## Introduction

Next generation DNA sequencing now allows large portions of the genomes of organisms to be screened for polymorphism and divergence during the speciation process (Hudson, 2008; Rokas and Abbot, 2009; Stapley et al., 2010). The upgrade in technology has made a key objective of evolutionary biology—understanding the genetic basis for trait variation, adaptation, and speciation—more accessible. Indeed, the upgrade has in many cases inverted the traditional approach from one that used to start with phenotypic variation of interest to one where diverged genomic regions of potential evolutionary significance are first identified and their phenotypic consequences subsequently determined (Feder et al., 2013; Nosil and Feder, 2013; Wray, 2013). Surveying a genome sequence thus provides a way to find loci of potential evolutionary interest (e.g., statistical outlier loci with elevated divergence), enabling questions concerning the numbers, types, and distribution of genetic changes involved in adaptation and speciation to be addressed.

However, our approaches to understanding population divergence still largely rest on a foundation of theoretical population genetics built around considering one or a few genes at a time in isolation from the rest of the genome. We therefore have a good understanding of how the core evolutionary forces of mutation, migration, selection, and drift should affect change and population divergence at the level of individual genes (Yeaman and Otto, 2011). The action of these processes on individual loci has also been documented empirically (Coyne and Orr, 2004; Gavrilets, 2004; Barrett and Hoekstra, 2011; Nosil, 2012). However, recent theory has challenged the view that speciation can be explained solely by understanding small numbers of loci having exceptional divergence and large fitness effects (Feder et al., 2012a,b; Flaxman et al., 2013, 2014). Indeed, genome scans of many species, such as sticklebacks (Jones et al., 2012), *Heliconius* butterflies (Heliconius Genome Consortium, 2012; Nadeau et al., 2012; Kronforst et al., 2013), whitefish (Renaut et al., 2012; Gagnaire et al., 2013), stick insects (Nosil et al., 2012a,b; Gompert et al., 2014; Soria-Carrasco et al., 2014), cichlids (Keller et al., 2013; Wagner et al., 2013), sunflowers (Andrew and Rieseberg, 2013; Renaut et al., 2014), and mosquitoes (Lawniczak et al., 2010; White et al., 2010), have implied that widespread differentiation across the genome may characterize even early stages of ecological divergence.

This is not to say that previous theory ignored the aggregate effects of many loci nested within a genome. For example, Barton, co-workers, and others have demonstrated general conditions under which the coupled effects of multiple loci can create strong barriers to gene flow (Barton, 1983; Barton and Bengtsson, 1986; Gavrilets, 2004; Barton and de Cara, 2009; Bierne et al., 2011). The role of chromosomal linkage and rearrangements in promoting this process have also been targets of theoretical analysis (Felsenstein, 1981; Burger and Akerman, 2011; Yeaman and Whitlock, 2011; Feder et al., 2012b; Via, 2012; Yeaman, 2013). However, while previous multilocus theory provides a very useful and important framework, explicit predictions have generally been restricted to small to moderate numbers of loci (<100) and/or limitations on the strength of selection, levels of migration, and breadth of the recombination map. Given the scale and scope of modern datasets, further work is thus needed to create a general, predictive theoretical framework for speciation that considers the aggregate effects of many simultaneously evolving genes arrayed in the genome. Indeed, such a whole genome perspective could potentially reveal underappreciated emergent processes contributing to speciation.

**Figure 1:**
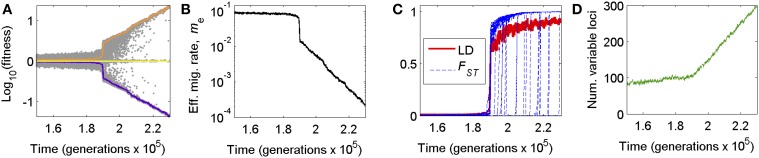
**Genome wide congealing can cause dramatic, nonlinear shifts in (A) local adaptation, (B) effective migration, (C) linkage disequilibrium and *F_ST_*, and (D) the rate of accumulation of divergently selected mutations. (A)** Average fitness of residents (orange) and immigrants (purple) over time relative to a randomly assembled genotype (yellow), depicting the rapid transition from genic to genomic phases of population divergence as GWC occurs. Gray dots are a random subsample of individual fitness values (200 individuals per generation sampled). **(B)** The effective migration rate, *m_e_*, as a measure of reproductive isolation arising from divergent local adaptation, during the transition into GWC. **(C)** The genome-wide average of linkage disequilibrium (LD) for pairs of loci on different chromosomes (single, red line) and *F_ST_* values (blue lines) over time for a random subsample of loci, depicting dramatic rises in these metrics of divergence accompanying GWC. **(D)** Jump in the rate of accumulation of divergently selected alleles as GWC occurs. Plots were produced from one example simulation run of the BU2S model (Methods; see also Flaxman et al., 2014) with *N* = 20,000 individuals, *m* = 0.1, *s* = 0.01, and no inverted regions in the genome.

As a step in this direction, we previously used forward-time, individual-based computer simulations (Flaxman et al., 2013, 2014) involving many mutations and incorporating genome structure to show how divergent selection and genome-wide linkage disequilibrium (LD) act in concert to split one population into two species once a threshold level of divergently selected mutations accumulate between populations undergoing gene flow (Figure 1). Selection on distinct suites of alleles reduces effective migration (*m_e_*) globally for the genome, resulting in greater differentiation for already diverged loci and increased probability of establishment of new mutations. This process can result in rapid, nonlinear transitions of one species into two. These transitions do not require particular mutations of large effect, but rather can occur simply by positive feedback between divergent selection and LD among many loci experiencing individually weak direct selection (Flaxman et al., 2014).

Consequently, the genomes of different populations begin to “congeal” into alternative, differentiated adaptive states representing reproductively isolated species (Figure 1). Even after the transition, there will still be heterogeneity among loci in levels of divergence because new mutations that arise after the transition still require time to become differentiated between populations (e.g., Figure 1C). Nonetheless, after the transition, reproductive isolation (RI) changes from being a characteristic of specific genes to a property of the entire genome (Feder et al., 2012a, 2013). We refer to this phenomenon as genome wide congealing (GWC). For clarity, we note that GWC leads to the kind of multilocus “coupling” that previous works (cited above) have described. However, the phenomenon we call “GWC” is more specific than “coupling” *per se*: GWC refers to a phase transition in speciation that is driven specifically by the positive feedback of genome wide divergent selection and linkage disequilibrium with each other.

A key point highlighted by GWC is that, when gene flow is significant, speciation can require indirect effects of loci on one another, and those effects are only possible with genomic structure (i.e., having genes together in genomes). Considered separately in isolation, each small-effect mutation would not overcome gene flow to attain a significant level of differentiation nor cause much RI between taxa; indeed, most polymorphisms would be expected to be transient. However, when a threshold number of such mutations eventually accumulate, their collective action can reduce the effective gene flow rate across the genome enough to allow all divergently selected alleles to undergo a marked jump in divergence and LD, increasing RI (Figure 1).

A key prediction of GWC is that two successive stages of speciation-with-gene-flow exist: (i) an initial “genic” phase in which differentiation is localized to isolated regions of the genome and predominately due to the direct effects of divergent selection acting on individual genes, and (ii) a subsequent “genomic” phase in which differentiation and RI become more genome-wide. Examples of these phases are seen, respectively, in Figure 1 in the periods before and after the transition from one population to two (mostly) reproductively isolated species. These phases have parallels to different concepts of genic vs. biological species (Wu, 2001). Populations residing in genic vs. genomic phases are predicted to display markedly different distributions in SNP allele frequency divergence, LD, and *F_ST_* (Figure 1).

A key driver of GWC is LD among divergently selected sites (Felsenstein, 1981; Barton, 2010). Thus, another prediction is that factors that reduce recombination in the genome (and thus help preserve LD) will promote GWC. One such factor is chromosomal rearrangements that invert the linear ordering of genes along chromosomes, i.e., “inversions” hereafter (Noor et al., 2001; Rieseberg, 2001). Here, we extend previous theory to consider the effects inversions may have on the dynamics of population divergence, with specific reference to GWC. Our work differs from past theory because although inversions have received much theoretical treatment (see below and Table 1), particularly concerning the mechanisms governing their initial spread and levels of genetic differentiation within them, the genome wide consequences of inversions for the dynamics of speciation are less clear.

**Table 1:** **Summary of genic inversion models since 2001, in terms of their consideration of: (i) the initial establishment or spread of the inversion, and (ii) levels of genetic differentiation within the inversion (especially with respect to those observed in collinear regions)**.

	**(i) Establishment or spread of inversion**	**(ii) Genetic differentiation within the inversion**	**Citation(s)**
1. Noor/Rieseberg verbal “recombination suppression” models	Established in allopatry; mechanism not specified	Upon secondary contact, populations remain more genetically differentiated for genes within inversions than for collinear regions (the latter being prone to homogenization)	Noor et al., 2001; Rieseberg, 2001
2. Navarro and Barton “accumulating differences” model	Mechanism not specified	DMIs can preferentially accumulate within inverted regions, relative to collinear ones, at least for some periods of the speciation process (assumes *s* >> *m_e_*)	Navarro and Barton, 2003a
3. Kirkpatrick and Barton “sympatric origins” model	A newly formed inversion captures locally adapted genes in hybridizing populations and is favored over the ancestral, collinear arrangement because the inversion keeps well-adapted genotypes intact; initial establishment of inversion is thus shown to be possible by natural selection and with migration/gene flow	Not focused on	Kirkpatrick and Barton 2006
4. Feder and Nosil “maintaining divergence” model	Established in allopatry; mechanism not specified	Upon secondary contact, populations remain more genetically differentiated for genes within inversions than for collinear regions, but only for a period of time and dependent on extensively reduced recombination in the inversion	Feder and Nosil, 2009
5. Feder and colleagues “mixed origins” model	Low frequency inversions persist in allopatric populations and contain the full complement of locally adapted genes due to lack of gene flow. Following secondary contact, the selective advantage of reduced recombination overpowers the slightly deleterious meiotic effects in heterokaryotypes, resulting in the adaptive spread of the inversion	Not focused on	Feder et al., 2011
6. Guerrero and colleagues “neutral expectations” model	Inversions established by different forms of selection and by drift were studied	Differentiation patterns of neutral loci within, across, and outside of inversions are examined under different selective regimes (inversion breakpoints themselves under selection vs. genes within them under selection)	Guerrero et al., 2012
7. Feder and colleagues “genome wide congealing” model	Inversions are pre-existing within populations and upon secondary contact the dynamics of their rise or fall in frequency are determined by the interplay of selection, gene flow, and drift	Maintenance and further build up of divergence in inversions following secondary contact is considered	This study

The logic behind a potential role for inversions in GWC is that inversions may facilitate transitions from genic to genomic phases because they can reduce rates of genetic exchange between heterokaryotypes (i.e., alternate chromosomal arrangements) several orders of magnitude below those for collinear regions, and if inversions are large this might affect substantial stretches of the genome (Noor et al., 2001; Rieseberg, 2001; Faria and Navarro, 2010). Thus, a number of loci can potentially be affected by “divergence hitchhiking” (Via, 2009, 2012) within an inversion, allowing the often-narrow window of reduced gene flow around a divergently selected site to be larger than in a collinear region (Feder et al., 2012b; Flaxman et al., 2012, 2013). While GWC does not require physical linkage of divergently selected loci, previous work has shown that linkage can, in some cases, accelerate the approach to GWC (Flaxman et al., 2014). Hence, by reducing recombination over larger stretches of the genome, inversions might magnify the effects of linkage and thereby accelerate population divergence.

Table 1 summarizes several key models on the role of inversions in divergence and speciation and highlights their foci with respect to the two key evolutionary issues determining the role of inversions in speciation: (i) the establishment and spread of inversions themselves, and (ii) the accumulation of genetic differentiation involving loci within inversions. Many earlier models (prior to 2001) assumed that new rearrangements had negative fitness consequences in heterokaryotypes due to meiotic irregularities they caused associated with single exchange events (reviewed by Rieseberg, 2001). Inversions were therefore considered to represent an example of underdominance. Thus, in most cases, inversions were presumed to be present in low frequency in populations due to mutation-purifying selection balance. Various scenarios involving meiotic drive, founder effects, and genetic drift were invoked to explain how new inversions elevated to high frequency and/or fixed in populations. However, as Rieseberg (2001, p. 351) noted, these early models lacked generality because “… the fixation of strongly underdominant chromosomal rearrangements through drift is unlikely, except in small, inbred populations.” The same logic about underdominance applies to meiotic drive since it often causes reductions in fertility (Crespi and Nosil, 2013). Indeed, mixed empirical evidence was found for strong meiotic reductions in fitness for many inversions segregating in populations (Faria and Navarro, 2010). Also, cases were found for inversions existing as high frequency polymorphisms in populations (e.g., Anderson et al., 1975). Hence, more recent “genic” models have focused on: (i) the fitness effects of the loci that rearrangements contain, and (ii) the recombination suppressing effects of inversions (Table 1).

In particular, Kirkpatrick and Barton's (2006) influential and important model showed that inversions arising in primary contact that captured favorable combinations of divergently selected alleles could spread in the face of gene flow and enhance differentiation between populations. The theoretical results generated much enthusiasm because of empirical examples reported in preceding years demonstrating enhanced divergence in inverted regions of the genome (e.g., Noor et al., 2001; Rieseberg, 2001), several involving ecological speciation (e.g., Feder et al., 2003a,b, 2005). However, one important caveat of the theory was that the newly arising inversion needed to capture essentially all of the segregating alleles favored in one of the two alternate habitats (populations) to establish. With modest gene flow generating departures from perfect adaptation within populations, the probability of this is not high, and even if it does occur the single copy of a new inversion would need to avoid stochastic loss by drift to establish. Feder et al. (2011) showed how inversion establishment in the face of gene flow could be promoted if inversions initially arose in allopatry. This was because allopatric populations are well adapted across the genome such that newly formed inversions had a high probability of containing locally adapted alleles, and because inversions can be present as multiple copies in allopatry, decreasing chances of stochastic loss. Consequently, following secondary contact of the allopatric demes and gene flow, selection for reduced recombination among the divergently adapted genes contained within the inversion will favor the inversion's spread in the population it originated in vs. retention of the ancestral arrangement in the alternate population.

Kirkpatrick and Barton's (2006) model therefore helped addressed what happens when an inversion captures loci important for divergence. However, it left several questions partially open, including: how often do these effects occur? How many more divergently selected mutations might subsequently accumulate prior to the completion of speciation (i.e., RI across the entire genome)? To what extent can inversions speed up speciation? Hence, the maintenance or accumulation of genetic differences within established inversions was the focus of other models (Navarro and Barton, 2003a; Feder and Nosil, 2009; Table 1). For example, Feder and Nosil (2009) modeled inversion dynamics following secondary contact for inversions that were initially fixed between allopatric populations. Although inversions could maintain elevated differentiation relative to collinear regions for some period of time following secondary contact for genes causing intrinsic post-zygotic isolation or those favorable in one habitat and neutral in others, even low levels of recombination in inversions resulted in inverted regions eventually exhibiting similar levels of divergence to collinear regions (although the time window of elevated divergence within inversions could be large in some cases). Moreover, divergence was not predicted to necessarily be concentrated (and by inference accumulate) in inversions. However, this model left open the question of how inversions arose and fixed in the first place. Thus, existing models generally examine the origins/spread of inversions or the evolution of additional differences within them, but not both (but see Guerrero et al., 2012). A quantitative treatment of both issues simultaneously is our goal here, in the context of GWC.

In sum, previous theory has shown that inversions may indeed have effects on divergence and speciation, especially when they happen to capture two or more divergently selected loci. What are needed are extensions of the theory that address the frequency with which such effects can generally be expected to occur, the magnitude of these effects, and their genome wide consequences (i.e., including for divergence of collinear regions). To accomplish these extensions, three key questions must be addressed: (i) Given that a new inversion may or may not initially capture any divergently selected genes, how often do we actually expect inversions to have effects on the dynamics of adaptive divergence? (ii) When an inversion is present, under what conditions and to what extent does it potentially serve as a “hot spot” or “island” for the subsequent accumulation of adaptive divergence? (iii) Under what conditions do inversions shorten the waiting time to the transition of populations from genic to genomic phases of speciation, and what is the magnitude of such effects when they occur?

To address these questions, we performed forward-time computer simulations in which populations were seeded with inversions encompassing differing amounts of the genome. We varied periods of allopatry (i.e., no gene flow) from zero generations (primary contact) to tens of thousands of generations. Then, upon gene flow and thousands of additional generations of subsequent evolution, inverted vs. collinear regions were compared to assess whether and to what extent the rearrangements: (i) increased the rate of evolution of RI, (ii) disproportionately accumulated new adaptive mutations, and (iii) displayed elevated levels of allele frequency divergence for selected loci.

Effects were only found to be both common and pronounced when three conditions were simultaneously satisfied: (i) alternative chromosomal arrangements were fixed in two populations upon secondary contact, (ii) inversions were large (e.g., encompassing 5% or more of the genome), and (iii) gene flow was strong relative to the per locus strength of divergent selection (i.e., the genetics of adaptive divergence was based on many genes having small effects on fitness in conditions unfavorable for the maintenance of polymorphisms). However, there are likely exceptions to our findings above when, for example, adaptation depends on strong epistatic fitness interactions between a few loci of major effect, which we did not examine here. In this case, inversions of small size that happen to capture these genes may have disproportionate consequences for divergence, particularly in instances of primary contact. We conclude by discussing the empirical challenges in showing that inversions play a central role in facilitating speciation with gene flow.

## Methods

Computer simulations were conducted using the BU2S (“build up to speciation”) simulation program (Flaxman et al., 2013), an individual-based computer simulation program that models speciation with gene flow for two populations residing in alternate habitats experiencing divergent ecological selection. Individual-based models provide a natural way to simulate (1) the discrete nature of biological organisms, genomes, and genes, and (2) relevant, realistic stochasticity inherent in evolutionary genetic processes arising from random mutation, recombination, and drift. We extended the most recently published version of BU2S (Flaxman et al., 2014) to incorporate inverted portions of chromosomes. A description of key features of the modeling approach is given below. Versions of simulation source code (C programming language) and associated Makefiles are publicly archived at http://sourceforge.net/projects/bu2s/files/. Additional technical details are given in the supporting information (*SI*).

A total of *N* (= 1000, 4000, or 20,000) diploid individuals were included in the simulations, equally divided in the two populations; population size had minor, quantitative effects, so results shown below are for *N* = 4000 unless otherwise noted. Selection was soft (population size was held constant) with symmetric, relative, per-locus fitness contributions of 1 + *s* (favored, homozygous genotype), 1 + 0.5 *s* (heterozygous) and 1 (disfavored, homozygous) in one population, and this scheme being reversed in the other to generate fitness tradeoffs between habitats. Each generation consisted of migration of individuals (when applicable), followed by fitness-weighted reproduction, mutation, and replacement of the parents by the offspring. Individual fitness was determined multiplicatively as the product of fitness contributions from all loci. Relative fitness determined the probability that an individual was chosen to be a parent for a reproduction event; the probabilistic nature of this process simultaneously incorporates selection and drift.

Simulations were begun with the populations geographically separated in allopatry with no gene flow (*m* = 0) for varying periods of time ranging from 0 to 50,000 generations prior to secondary contact. As alluded to above, the initial period of allopatry allowed for populations to potentially accumulate a degree of differentiation unopposed by gene flow and for inversions to potentially contain a block of such divergently selected loci prior to secondary contact (conditions shown by Feder et al. (2011) to be most favorable for the differential establishment of a new inversion). To do this, secondary contact began with the alternate chromosomal arrangements at one of two frequency distributions: (i) an initial low frequency of one or more inversions, 0% in one population and 2% in the other population, or (ii) local fixation of one or more inversions, 0% in one population and 100% in the other (here and subsequently, frequencies refer to inversions, i.e., derived, inverted arrangements). Only these two extremes were explored in order to keep the number of parameter combinations manageable.

We modeled genomes having a total recombination length of 1000 centi-Morgans (cM) equally divided into 10 chromosomes (i.e., diploid 2*n* = 20 chromosomes; each chromosome 100 cM long). These numbers were chosen to represent a generic organism since many species have numbers of chromosomes on the order of 10^1^ and chromosomes that undergo 1–2 synapses per meiosis (general effects of genome size in the BU2S model were explored by Flaxman et al., 2014). To most easily reveal the consequences of inversions, we focused on large inversions encompassing 50 cM of a chromosome (i.e., half of one chromosome), and we added from zero to five such inversions to replicate simulations that were identical in all respects except for the numbers of inversions added. We used these relatively large inversions not because they are reflective of some average size found in nature but rather because preliminary simulations indicated that using smaller inversions simply resulted in quantitatively smaller effects or the absence of effects (Figures S1, S2). Stochastic recombination events involving collinear arrangements were modeled as being independently identically distributed with a mean of 50 cM between recombination points. For heterokaryotypic individuals, recombination between inverted and noninverted portions of a chromosome were allowed to occur with a probability of 10^−8^ per gamete and an equal gene-for-gene exchange was assumed (i.e., a double recombination event was required and gametes were “balanced” in terms of their genetic content). We imposed a mild fitness cost, a 0.1% reduction, on heterokaryotypic individuals.

New divergently selected mutations were added uniformly and randomly across the genome—i.e., with the same probability, per cM, to inverted and collinear regions—through the course of a simulation run at a rate of one mutation per generation (consistent with rates of adaptive mutations from empirical studies: Halligan and Keightley, 2009). Selection coefficients were drawn from an exponential distribution with mean *s*. This, combined with our multiplicative fitness scheme (above), assumes that fitness consequences of mutations are constant over much evolutionary time (rather than quickly diminishing in magnitude as some sort of environmental optimum is approached). Such a scheme could realistically represent evolution in environments that are consistently changing and/or have many dimensions for adaptation. Upon secondary contact, individuals migrated to the other deme with probability *m* per individual per generation. A range of *s* (= 0.005, 0.01, or 0.02) and *m* (= 0.001, 0.002, 0.005, 0.01, 0.02, 0.05, or 0.1) combinations were used; the most revealing combinations for cases with and without effects of inversions are shown below and in the *SI*.

The simulations were continued after secondary contact either for a total of 1.2 million generations or until a threshold barrier strength (sensu Barton and Bengtsson, 1986) of *m*/*m_e_*≥500 was attained. The barrier strength was therefore a measure of the reduction in effective gene flow across the genome (i.e., a proxy for RI). The barrier strength of 500 was chosen because it represented extremely reduced gene flow but, for simulations that would reach GWC, it was still highly feasible to run simulations long enough to reach this barrier. Most importantly, this provided an objective way to compare waiting times to a given (arbitrary but strong) divergence point across different simulations and parameter combinations.

The simulations have a great deal of biological realism, but necessarily leave out more than they include. Especially relevant in the context of speciation is that our results were generated without epistasis or intrinsic incompatibilities, which are, of course, of great importance in general in speciation, perhaps especially for the latter stages of speciation (Coyne and Orr, 2004; Gavrilets, 2004; Nosil, 2012). We left out epistasis and incompatibilities because, in the context of inversions, previous hypotheses have focused extensively on how reduced recombination and effective gene flow might promote speciation (Table 1). Hence, our simulations were designed around that issue. By leaving out other factors, we are able to isolate the importance of recombination reduction *per se*. Future work on epistasis is certainly warranted (see Discussion).

## Results

**Figure 2:**
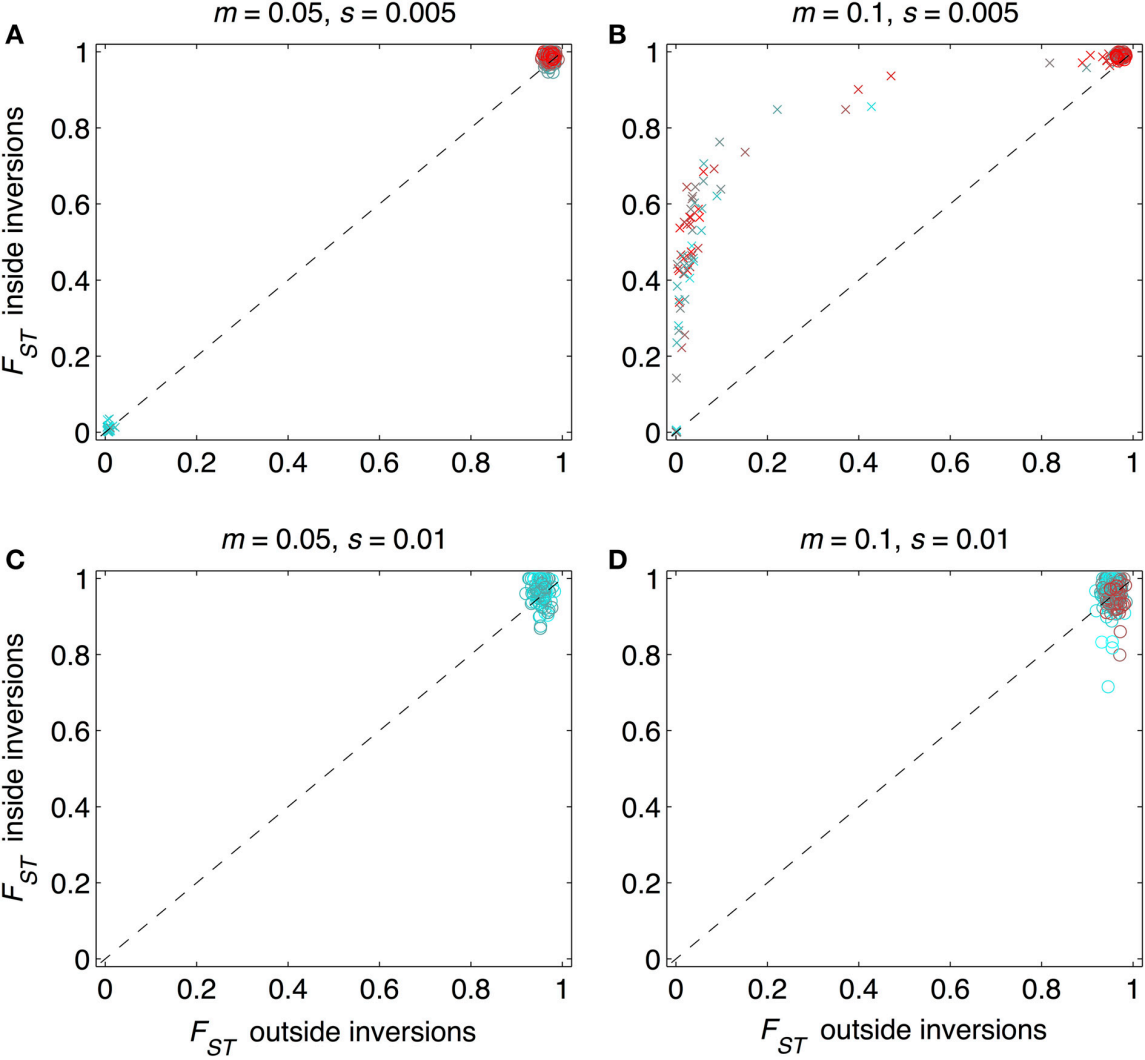
**Mean *F_ST_* values for loci within and outside inverted regions for 984 simulation runs**. Circles show runs in which divergence reached a designated barrier strength, *m*/*m_e_*≥500; “x” symbols show runs that did not reach this barrier strength within the allotted time (1,200,000 mutations and generations). Periods of allopatry were varied in steps of 1000 generations. As colors change from cyan to red, the period of allopatry changes, respectively, from zero to as long as 50,000 generations (when red symbols are not visible, e.g., in **(C)**, it is because the barrier was reached even prior to the end of the allopatric period). The 1:1 line is the null expectation that mean *F_ST_* for loci within inverted regions would be the same as those outside inversions. Each point shown is from a different simulation run. Combinations of the gross migration rate, *m*, and the average per locus strength of divergent selection, *s*, are given above each panel: **(A)** *m* = 0.05, *s* = 0.005; **(B)** *m* = 0.1, *s* = 0.005, **(C)** *m* = 0.05, *s* = 0.01, **(D)** *m* = 0.1, *s* = 0.01.

**Figure 3:**
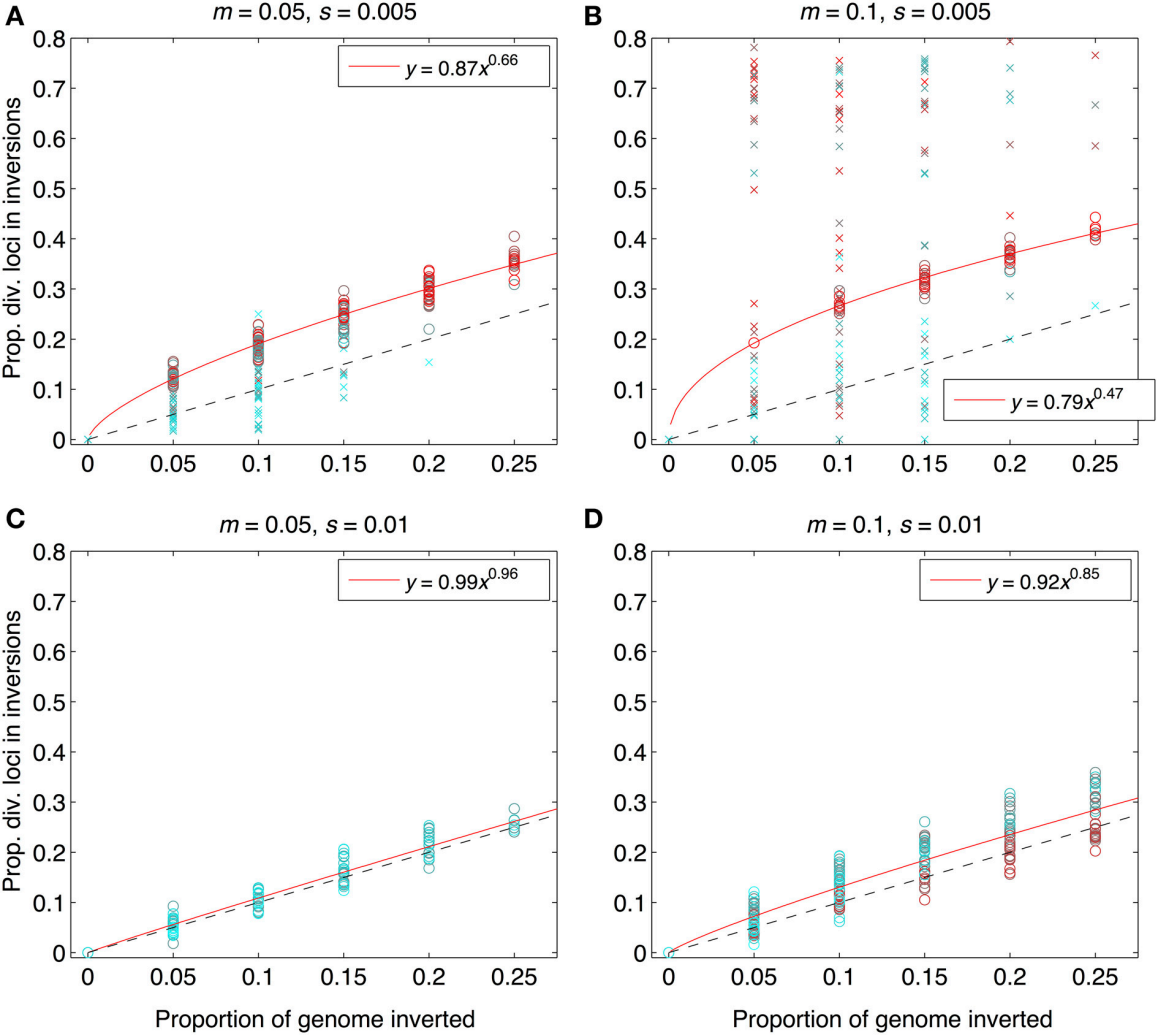
**Proportions of divergent loci found within inverted regions for the same set of simulation runs as in Figure 2**. The x-axis shows the proportion of the genome with segregating inversions; the y-axis shows the proportion of divergent loci located within inversions. Both were measured at the end of each of 984 simulation runs. Interpretation of symbol shapes and colors is the same as in Figure 2. The black, dashed line is the null expectation (1:1) if inversions have no effect. Solid, red lines are fits from power law regression (equations in legend within each panel) through the subset of points from those runs that reached the designiated barrier strength (i.e., the points represented by open circles), forced through the origin. For numbers of polymorphic loci (rather than proportions), see Figure S3. Parameter combinations are the same as in **Figure 2**: **(A)** *m* = 0.05, *s* = 0.005; **(B)** *m* = 0.1, *s* = 0.005, **(C)** *m* = 0.05, *s* = 0.01, **(D)** *m* = 0.1, *s* = 0.01.

**Figure 4:**
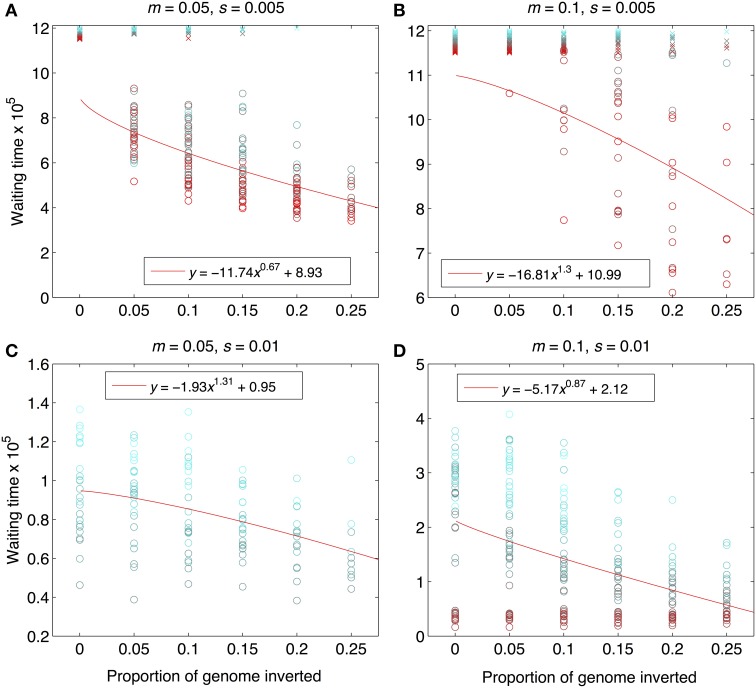
**Waiting times to reach the designated barrier strength, *m*/*m_e_*≥500**. Data are from the same set of simulation runs as Figures 2, 3, and the interpretation of the symbols and the solid, red lines are the same. The “waiting time” was calculated as the number of generations that elapsed between the end of the period of allopatry and the time when the barrier strength was reached. Note that y-axis scaling differs across panels in order to maximize visual resolution. Parameter combinations are the same as in **Figure 2**: **(A)** *m* = 0.05, *s* = 0.005; **(B)** *m* = 0.1, *s* = 0.005, **(C)** *m* = 0.05, *s* = 0.01, **(D)** *m* = 0.1, *s* = 0.01.

As expected from previous work, under some conditions inversions could help accelerate the speciation process (Figures 2–4). Specifically, inversions had the most pronounced consequences for divergence when the average effect size of mutations was small relative to the gross migration rate (e.g., *s*:*m* = 1:20 in Figure 2B). This was true regardless of whether the metric for divergence was the *F_ST_* values of diverged loci in inverted vs. collinear regions (Figure 2), the excess proportion of differentiated loci in inverted vs. collinear regions (Figure 3), the time it took to reach the threshold barrier strength (Figure 4), or the proportion of simulations runs reaching the barrier strength prior to 1.2 million generations (compare relative amounts and locations of “x” and “o” symbols in Figures 2–4).

However, even under parameter combinations of *m* and *s* that maximized the effects of inversions, these effects were not necessarily expected to be common; a major factor determining how often inversions accelerated divergence was the magnitude of inversion frequency differences between populations at the onset of gene flow. For the parameter values we explored, pronounced effects of inversions were only common when inversions were alternatively fixed between populations upon secondary contact (compare Figures 2–4 to Figures S4–S6). Inversions initialized at a frequency of 2% were very frequently lost from populations (>90% of inversions were lost in Figures S4–S6). As a result, simulations starting with inversions at low frequency at secondary contact often failed to find an appreciable impact of inversions for divergence (Figures S1–S6), simply because the large majority of inversions were lost. It is worth noting, however, that on the rare occasions that these initially small frequency-inversions established, they reliably resulted in shorter waiting times and a higher proportion of divergent loci within inversions (relative to the null expectation).

We further note that the results shown in Figures 2–4 were the conditions in our simulations that maximized the potential for inversions to have effects on promoting divergence. First, the simulations represented large inversions that subsumed a fair proportion of the genome, increasing the chances that at least some divergently selected mutations would be contained therein; results with smaller inversions were much less likely to reveal effects (Figures S1, S2). Second, we considered ranges of time periods of allopatry that, for displayed values of *s* and *m*, were usually insufficient for the evolution of substantial levels of RI prior to secondary contact. Longer periods of time will, of course, enable the buildup of more differences between diverging populations, such that they may pass the point of GWC (i.e., “speciate”) before secondary contact, independently of the presence or absence of inversions (Figure S7). In such cases, recombination reductions from inversions will again have little to no role in the dynamics of speciation. However, periods of allopatry that are too short will generally make it less likely for inversions to capture loci generating RI, since there hasn't been enough time for such loci to evolve (Figure S7).

**Figure 5:**
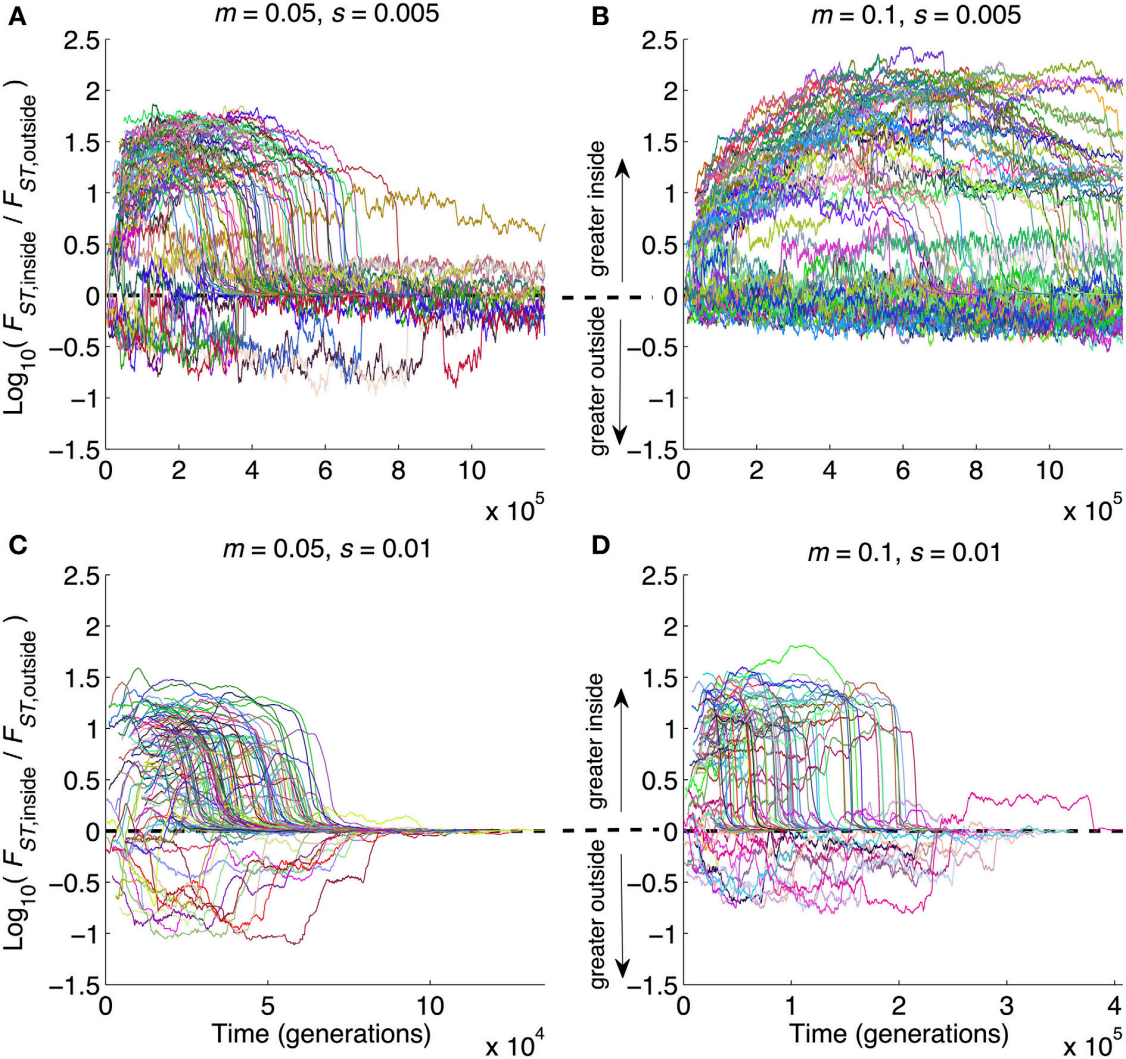
**Time series of average divergence at sites inside inversions relative to outside inversions**. Data are from the same set of simulation runs as Figures 2–4, with parameter combinations noted above each panel. Whereas those figures show results at the ends of runs, here the results are time series from the beginning to end of each run. Each run is a separate line. At each point in time, the value of the line is the ratio of mean *F_ST_* for polymorphic sites inside inversions divided by mean *F_ST_* of polymorphic sites outside inversions. Note that the y-axes are Log_10_-transformed, and the x-axes vary in scaling from panel to panel in order to provide maximum visual resolution. Parameter combinations are the same as in **Figure 2**: **(A)** *m* = 0.05, *s* = 0.005; **(B)** *m* = 0.1, *s* = 0.005, **(C)** *m* = 0.05, *s* = 0.01, **(D)** *m* = 0.1, *s* = 0.01.

One of the advantages offered by our modeling approach is that, in addition to looking at patterns at a given point in time (as in Figures 2–4), we could also follow the dynamics of divergence over time from start (zero divergence) to finish (strong multilocus barriers to gene flow and RI). Figure 5 shows how divergence built up inside relative to outside of inversions as the ratio Log_10_(*F*_*ST*,inside_/*F*_*ST*,outside_). This metric can obviously only be calculated when inversions are still segregating and when at least one polymorphic site occurs in an inversion. Hence, caution must be taken when interpreting Figure 5 because it does not display results from the many runs in which inversions were lost from the populations. With that in mind, several points are notable. First, all four panels show at least some runs with the same qualitative pattern: divergence can be widely different inside vs. outside of inversions prior to GWC, and then when GWC is reached, divergence becomes more uniform (i.e., the ratio (*F*_*ST*,inside_/*F*_*ST*,outside_) approaches 1, causing Log_10_(*F*_*ST*,inside_/*F*_*ST*,outside_) to approach 0). This pattern also emphasizes the temporally transient nature of elevated divergence: even when inversions speed up divergence, detecting strongly elevated divergence within inversions may be contingent upon happening to study a pair of populations at the right time. Second, the parameter combinations with the biggest effects of inversions on other metrics (Figures 2B, 3B, 4B) showed the greatest amount and longest duration of elevated ratios (Figure 5B). Third, there were many cases in which divergence was actually lower inside inversions than outside of inversions (lines dropping below 0 in all panels of Figure 5). Hence, even when the parameters that characterize a population are favorable for inversions to promote divergence, stochastic events—rare recombination in inverted regions, large *s* mutations in collinear portions of the genome—can cause the opposite pattern to be observed. This underscores that the role of inversions in a given case of divergence may be nuanced and dependent upon stochastic mutation and recombination events. Unfortunately, this perhaps complicates the interpretation of “snapshot” empirical patterns based on divergence at a single time point. This also serves as a very strong reminder of the value of conducting many replicates of stochastic simulations; simply examining one run or central tendencies would have produced a much different interpretation of Figure 5.

## Discussion

We investigated three questions about the role of inversions during adaptive divergence of populations: (i) Given that a new inversion may or may not initially capture any divergently selected genes, how often do we actually expect inversions to have effects on the dynamics of adaptive divergence? (ii) When an inversion is present, under what conditions and to what extent does it potentially serve as a “hot spot” or “island” for the subsequent accumulation of adaptive divergence? (iii) Under what conditions do inversions shorten the waiting time to the transition of populations from genic to genomic phases of speciation, and what is the magnitude of such effects when they occur? Some of the features that distinguished our approach from previous works included that we did not force inversions to contain any specific number of divergently selected loci, we allowed the buildup of divergence for variable amounts of time both before and after inversions arose, and we explored evolutionary trajectories from different population frequencies of segregating inversions.

We found that inversions can certainly have strong effects on divergence dynamics, but for the range of scenarios we considered, these effects are expected when *s* << *m*, there is a “Goldilocks” period of allopatry (not too short and not too long), and inversions are large and encompass a significant portion of the genome. And even under such conditions, we have the further caveat that inversions are unlikely to persist (and be able to affect speciation) unless inversion frequency differences between populations are large upon secondary contact. Thus, the role of inversions in promoting genome wide divergence during speciation is not expected to be ubiquitous. The latter point is underscored by the observation of patterns of reduced divergence in inversions in some instances (lines below 0 in Figure 5).

However, in the right circumstances, inversions can potentially make a large contribution. The “right circumstances” are certainly biologically plausible in many systems, given that both theory and data suggest most new adaptive mutations will have small effects on fitness, and adaptive traits that show more continuous patterns of variation are frequently controlled by a complex and polygenic genetic architecture (e.g., Fisher, 1930; Fishman et al., 2002; Valdar et al., 2006; Buckler et al., 2009; Flint and Mackay, 2009; Huang et al., 2009; Brachi et al., 2010; Chan et al., 2011; Tian et al., 2011; Hung et al., 2012). Navarro and Barton (2003a,b) have also shown that inversions can aid in the establishment of mutations having small effects on performance but increasing post-mating RI between populations (Bateson-Dobzhansky-Muller incompatibilities). However, in this instance *m_e_* levels must already be substantially reduced between populations for the new mutations to accumulate and strengthen the barrier.

Qualitatively, many of our results were consistent with previous models of inversions (Table 1). Our results for inversions also mirrored previous findings for the effects of divergence hitchhiking (DH) on speciation (Feder et al., 2012b; Flaxman et al., 2012, 2013, 2014; Yeaman, 2013). In retrospect, this is not unexpected, as inversions essentially increase the window of opportunity for DH to act, and by assumption, we focused on the recombination-suppressing effects that inversions have. When effect sizes for new mutations are relatively high, individual mutations can establish on their own without the need for genetic hitchhiking associated with genome structure. But as (i) the effect size of adaptive mutations is reduced, and/or (ii) inversions subsume a greater proportion of the genome, rearrangements can have substantial quantitative effects on the genomic distribution of diverged loci and the waiting time to speciation.

Our work is set apart from most previous works in several ways. First, we considered the buildup of divergently selected alleles dynamically for varying periods of time prior to and after the origin of inversions. Second, we made no assumptions forcing inversion to contain any loci important for divergence and local adaptation. Third, our individual-based modeling approach simultaneously integrates selection, drift, recombination, and gene flow. The combination of these features of our work allowed us to make quantitative predictions about conditions under which inversions affect the dynamics of speciation with gene flow and the magnitudes of these effects. These quantitative predictions include predictions about what fraction of divergently selected loci should be found inside inversions, how much inversions can speed up speciation in a given parameter scenario, and the extent to which loci inside inversions will be more differentiated than loci outside the inversions. Predictions about temporal trajectories of divergence metrics (Figure 5) are also a unique feature of our work. In aggregate, these temporal trajectories give a very clear rationale for why different studies may find different results about inversions in their respective systems: even in the same conditions, inversions may sometimes speed up and sometimes slow down divergence. That is a challenging result from the perspective of empirical testing, but one that is still valuable; without such knowledge, investigators are likely to see conflicts between ideas or results when in fact there is none.

### Extensions of the theory: underdominance and epistasis

Our results included only weak underdominance (0.1% reduction in fitness in heterokaryotypes). This choice was made based upon theoretical and empirical considerations (Rieseberg, 2001), as noted above. Nonetheless, if strong underdominance occurred upon secondary contact, we expect that this might accentuate the roles of inversions in speeding the approach to GWC. Hence, exploring the relationship between underdominance and the dynamics of GWC is likely to be a worthwhile endeavor for future theory. It will also likely prove useful to explore additional parameter ranges for inversion frequency distributions, numbers and sizes of chromosomes in the genome, inversion sizes, and mutation effect size distributions.

Like Kirkpatrick and Barton's (2006) model, our models did not involve epistasis. Thus, considering how epistatic interactions between loci might help inversions drive GWC would certainly be a useful extension for future investigations, especially given that epistasis can be pervasive in evolution (Breen et al., 2012). However, the same considerations should apply with respect to DH: inversions create larger windows for epistatic mutations to arise and accumulate in the face of gene flow (Navarro and Barton, 2003a). Consequently, when adaptive evolution involves epistatic fitness interactions among loci of relatively large effect, as suggested by some recent empirical studies (Wilfert and Schmid-Hempel, 2008; Breen et al., 2012), then inversions that happen to capture such loci could make a quantitative difference in facilitating divergence with gene flow.

Thus, if epistasis is common, inversions could play a larger role in speciation than suggested here by increasing the chances that new mutations arise in tight linkage with and, during critical stages of their initial establishment, are kept in the same phase with already diverged complementary loci. But we note that new mutations can often arise in disfavored genetic or ecological backgrounds, and in such cases reduced recombination can actually hinder mutation establishment (Hill and Robertson, 1966; Feder et al., 2012b). This is simply a specific example of why recombination is so common (Maynard Smith, 1977; Ortiz-Barrientos et al., 2002). Inversions arising in primary contact that capture favorably interacting epistatic suites of genes may also have higher probabilities of establishment than is generally the case. However, again the limitation applies that the inversion must generally capture all favorably interacting alleles together. Thus, most such inversions will likely be smaller in size (though smaller inversions may also suppress recombination more effectively).

### Final thoughts and conclusions

We have discussed how chromosomal rearrangements can potentially facilitate speciation with gene flow in two inter-related ways. One way is through the inversion capturing co-adapted suites of alleles and protecting them from recombinational breakdown in the face of gene flow. Once such complexes have become established, they could potentially serve as prepackaged sources of standing variation when ecological opportunity presents itself. The second way is through inversions accumulating additional divergently selected loci at an accelerated rate compared to collinear regions of the genome. Our forward-time simulations included the potential for both.

With respect to inversion origins, we discussed how cycles of allopatry and secondary contact are generally more favorable for the establishment and spread of new rearrangements than cases of primary contact. However, it is still possible for inversions to arise and spread in primary parapatry and sympatry (Kirkpatrick and Barton 2006). With respect to fostering an increased rate of adaptive substitution, inversions can be thought of as widening the window that divergence hitchhiking (DH) could potentially act upon. It has been argued that DH makes divergence with gene flow much easier (Via, 2009, 2012). However, recent work shows that this is not always the case, and the issue is more complex and nuanced (Feder and Nosil, 2010; Feder et al., 2012b; Flaxman et al., 2012, 2013, 2014; Yeaman, 2013). In this regard, an important transition in speciation with gene flow is the phase shift from genic to genomic divergence enabled by genome wide reductions in effective migration that trigger GWC (Feder et al., 2012a, 2013; Flaxman et al., 2014). DH can play a role in speeding passage through the genic phase of speciation by creating nucleation points of local or regional congealing in the genome. From the perspective of facilitating speciation with gene flow, inversions are essentially a form of DH in which the size of the chromosomal region over which linkage of a new mutation to an already diverged gene is enlarged, due to reduced recombination in heterokaryotypes. Thus, inversions will increase the potential for DH to act. However, as our computer simulations demonstrated, the same scenario of *s* << *m* being required for genome structure to matter for speciation applies.

Empirical verification of these predictions will be difficult, for example due to the complex interplay of the processes affecting the origins and spread of inversions vs. differential build-up of divergence in them. Different processes might leave similar patterns (Guerrero et al., 2012). Nonetheless, progress can be made. For example, the ages of the inversions and the co-adaptive genes they may contain are key pieces of information. Breakpoints of inversions and the immediate flanking collinear regions generally have extremely low exchange rates. As such, comparisons of sequences near chromosomal breakpoints would be expected to provide information about the age of a rearrangement, which can serve as a foundation for hypothesis testing (e.g., White et al., 2009; Cheng et al., 2012).

When testing the geographic origin hypotheses for the establishment and spread of inversions, it is expected that if a rearrangement arose during a period of allopatry, then the level of divergence in the breakpoint regions for the inversions should approximate the time of geographic separation of the two populations. In contrast, inversions originating in sympatry should be younger. And if the current area of geographic overlap between taxa represents a zone of secondary contact, then there should be sequences in other regions of low recombination in the genome that appear older than the breakpoints. The difficulty then involves determining whether haplotypes can be found in sympatrically arising inversions that significantly predate the age of the inversion, and by implication, were captured by the rearrangement when it arose. In the allopatric scenario, adaptive haplotypes within the inversion and the breakpoints may not display great differences in their apparent ages due to both sequences accumulating divergence from the time of allopatry.

When testing for inversions accelerating the rate of adaptive evolution, it should be shown that a significantly greater number of new derived mutations of younger age than the breakpoints accumulated in the rearrangements compared to collinear regions. Given the existence of the inversion polymorphism in an outgroup taxa or population to serve as a point of reference to assign the ancestral vs. derived status of variants, it may be possible to test for an increased rate of nonsynonymous substitutions in the lineages of the “in-group” taxa, particularly if one of the populations inhabits a novel environment compared to the outgroup and divergence is old enough. Such a finding would support DH, although a relaxation of selection pressures for the in-group taxa would still have to be ruled out.

In conclusion, inversions can play a contributing role in speciation with gene flow. However, enthusiasm concerning rearrangements stemming from initial empirical studies must be tempered to some degree by the realization that the effects of inversions are predicted to be more quantitative than qualitative and to be pronounced only under certain conditions. Indeed, new data for certain model organisms have demonstrated a more nuanced role for inversions in adaptive divergence and RI than originally envisioned (Strasburg et al., 2009; Michel et al., 2010). Specifically, genome scans have found numerous differentiated regions mapping outside chromosomal rearrangements (Strasburg et al., 2009; Jones et al., 2012; Reidenbach et al., 2012), although findings from *Drosophila*, *Mimulus*, *Heliconius*, and crows may provide exceptions (Noor et al., 2001, 2007; Lowry and Willis, 2010; Joron et al., 2011; Stevison et al., 2011; McGaugh and Noor, 2012; Poelstra et al., 2014). We are therefore gaining a clearer and more accurate understanding of the adaptive significance of inversions for population divergence and speciation.

With those points noted, we also emphasize that our approach considered only the recombination-suppressing effects of inversions. Rearrangements could have more prominent, qualitative effects if they cause changes in gene regulation. Additionally, while we sought to find areas of parameter space where inversions had the largest effects, the parameter space is vast and merits continued explorations. In sum, important theoretical extensions remain to be carried out, and empirical testing will involve finding clever new approaches to an intricate and complex problem.

## Author contributions

Jeffrey L. Feder, Patrik Nosil, and Samuel M. Flaxman conceptualized ideas, designed models, interpreted data, and wrote the paper. Samuel M. Flaxman programmed simulations.

### Conflict of interest statement

The authors declare that the research was conducted in the absence of any commercial or financial relationships that could be construed as a potential conflict of interest.
