# Supplementary material for: Assessing when chromosomal rearrangements affect the dynamics of speciation: implications from computer simulations
Source: Front Genet. 2014 Aug 26;5:295. doi: 10.3389/fgene.2014.00295 (PMC4144205; doi:10.3389/fgene.2014.00295)
Supplement: Supplementary file 1 [file DataSheet1.DOCX]

**Supporting Information (*SI*) for
“Assessing when Chromosomal Rearrangements Affect the Dynamics of Speciation: Implications from Computer Simulations”**by J. L. Feder, P. Nosil, and S. M. Flaxman

***SI* comprises:**

Supplemental Methods, giving additional details of simulation modeling

Figures S1 – S7

SUPPLEMENTAL METHODS

We used simulations to explore the *de novo* buildup of population divergence in a two-deme environment with divergent selection. Reproductive isolation (RI) occurred due to divergent local adaptation (i.e., “extrinsic” isolation), and was quantified by the strength of the barrier, *b*, to gene flow between demes. This barrier strength was calculated as *b* = *m*/*m*e (sensu Barton and Bengtsson, 1986), where *m* is the gross migration rate between demes and *m*e denotes the expected effective backward migration rate ([Vuilleumier et al., 2010](#_ENREF_9)), defined as the expected proportion of reproduction in a deme attributable to migrants. Populations consisted of *N* discrete individuals each having their own, potentially unique, explicit diploid genomes subject to soft selection and regulation of total population size (constant *N*). Evolutionary dynamics resulted from the combination of mutation, selection, migration, recombination, and drift.

*Individual-based modeling*

We used an individual-based model built upon a previously published computer program referred to as “BU2S” (“Build Up to Speciation”) by [Flaxman et al. (2013](#_ENREF_4)) and [Flaxman et al. (2014](#_ENREF_5)). Source code is available at http://sourceforge.net/projects/bu2s/. Our novel extensions consisted of adding code to enable consideration of reduced recombination arising from portions of chromosomes being inverted. We first describe the general workings of the model, and then how inversions were handled specifically in this context.

BU2S simulates the evolution of a finite population in a spatially heterogeneous environment. Individuals migrate between demes with probability *m* per individual per generation. A divergently selected mutation arises in a randomly chosen individual in a randomly chosen deme once per generation, consistent with empirically observed beneficial mutation rates ([Halligan and Keightley, 2009](#_ENREF_7)). The selection coefficient for the *j*th locus was denoted *sj* and was drawn from an exponential distribution with mean *s*. The contribution to fitness of each locus was similar to schemes used by [Felsenstein (1981](#_ENREF_3)) and Barton and de Cara ([2009](#_ENREF_2)): the fitness contribution of a locus was 1 + *sj* if homozygous for the favored allele, 1 + 0.5*sj* if it was heterozygous, and 1 if homozygous for the disfavored allele. The fitness of an individual *i* in deme *k* (*Wik*) was calculated multiplicatively as the product of contributions of all individual loci: , where *L* is the total number of divergently selected loci with two alleles segregating, and *wjk­* is the fitness contribution of locus *j* to the individual’s fitness given its genotype at that locus (*gij*) and its current deme (*k*).

Simulations were run for 1,200,000 generations—the maximum possible run time on the supercomputer for the slowest running parameter combinations—or until an *a priori* strength of the barrier to gene flow, *b*, (= *m*/*me*; sensu Barton and Bengtson 1986)was reached, whichever came first. In all results shown, we used *b* ≥ 500 as the operational RI threshold. Average, genome-wide linkage disequilibrium (LD) shown in Figure 1 was calculated over all possible polymorphic pairs of loci as the average LD correlation coefficient ([i.e., the “correlation of allelic states”: Freeman and Herron, 2004](#_ENREF_6)) for each pair of loci, $\frac{D}{\sqrt{p_{i}q_{i}p_{j}q_{j}}}$, where *D* is the standard coefficient of linkage disequilibrium, *i* and *j* are any two polymorphic loci, *pi* is the frequency of a given allele at locus *i*, and *qi* is the frequency of the other allele (= 1 – *pi*). We calculated single-locus *FST* values using the standard formula , where *HT* was the total observed heterozygosity at a given locus at a given time step and *HS* was the expected heterozygosity based upon each deme’s observed heterozygosity ([Hartl and Clark, 2007](#_ENREF_8)).

Gametes were produced assuming that allelic combinations on a given chromosome could only be broken up by recombination events. In collinear regions, recombination events were independently identically distributed with a mean of 50 centi-Morgans (cM) between consecutive events. Genomes had total map length *M =* 1000 cM and *C* = 10 chromosomes (i.e., each chromosome was 100 cM long).

*Adding inversions to chromosomes and populations*

In the results shown here and in the main text, the chromosomal location for an inversion was chosen randomly, with the constraint that two inverted regions could not be overlapping. The sizes of inversions were a specified parameter held constant within a simulation run (50 cM each in Figures 2-4, S4-S7; 10 cM each in Figures S1-S2). Inversions were considered to have only two effects in our simulations, and both of these effects only occurred in heterokaryotypic individuals: (i) reducing recombination (see below) and (ii) reducing individual fitness by 0.1%.

As noted in the main text and results, we added varying numbers of different inversions to populations at the time in a simulation when secondary contact commenced (0-5 inversions in Figures 2-4, S4-S7; 0, 1, 2, 5, or 10 inversions in Figures S1-S2). This enabled us to (i) evolve replicate simulated populations up to the same exact starting points prior to adding inversions and (ii) have inversions exist at a specified frequency upon secondary contact. When added, inversions were only added to individuals in one of the demes. If the designated frequency of an inversion was *p*, then a proportion *p* of all copies of the relevant chromosome in one deme were chosen at random to have that inversion. Results in the main text use *p* = 1, simulating a fixed inversion in one deme. Results here use *p* = 1 (Figure S7) or *p* = 0.02 (Figures S1-S6).

*Recombination involving inverted regions in heterokaryotypes*

In heterokaryotypic individuals, recombination still operated as described above over all parts of the genome that were collinear. However, in the region(s) encompassed by an inversion(s) in heterokaryotypic individuals, recombination was handled with the following rules. First, recombination was not allowed at all for a small span, 0.1 cM, around the breakpoints of an inversion. Second, the probability of recombination between alternative arrangements was 10-8 (i.e., very rare). Third, if a recombination event occurred between alternative arrangements, we required that it was a double-recombination event so that there could be an equal gene-for-gene exchange, leading to the production of genetically balanced gametes. The two breakpoints defining such a recombination event were chosen randomly (though obviously had to both lie within the inversion).

*References cited in Supplemental Methods*

Barton, N.H., and Bengtsson, B.O. (1986). The barrier to genetic exchange between hybridizing populations. *Heredity* 57**,** 357-376.

Barton, N.H., and De Cara, M.a.R. (2009). The evolution of strong reproductive isolation. *Evolution* 63**,** 1171-1190. doi: 10.1111/j.1558-5646.2009.00622.x.

Felsenstein, J. (1981). Skepticism towards Santa Rosalia, or why are there so few kinds of animals? *Evolution* 35**,** 124-138.

Flaxman, S.M., Feder, J.L., and Nosil, P. (2013). Genetic hitchhiking and the dynamic buildup of genomic divergence during speciation with gene flow. *Evolution* 67**,** 2577-2591. doi: 10.1111/evo.12055.

Flaxman, S.M., Wacholder, A.C., Feder, J.L., and Nosil, P. (2014). Theoretical models of the influence of genomic architecture on the dynamics of speciation. *Mol. Ecol.***,** online ahead of print, DOI: 10.1111/mec.12750.

Freeman, S., and Herron, J.C. (2004). *Evolutionary Analysis.* Upper Saddle River, NJ: Pearson Education, Inc.

Halligan, D.L., and Keightley, P.D. (2009). Spontaneous mutation accumulation studies in evolutionary genetics. *Annu. Rev. Ecol. Evol. Syst.* 40**,** 151-172. doi: 10.1146/annurev.ecolsys.39.110707.173437.

Hartl, D.L., and Clark, A.G. (2007). *Principles of population genetics.* Sunderland, MA: Sinauer Associates, Inc.

Vuilleumier, S., Goudet, J., and Perrin, N. (2010). Evolution in heterogeneous populations: From migration models to fixation probabilities. *Theor. Popul. Biol.* 78**,** 250-258. doi: 10.1016/j.tpb.2010.08.004.

SUPPLEMENTAL FIGURES S1-S7
(legends below each)


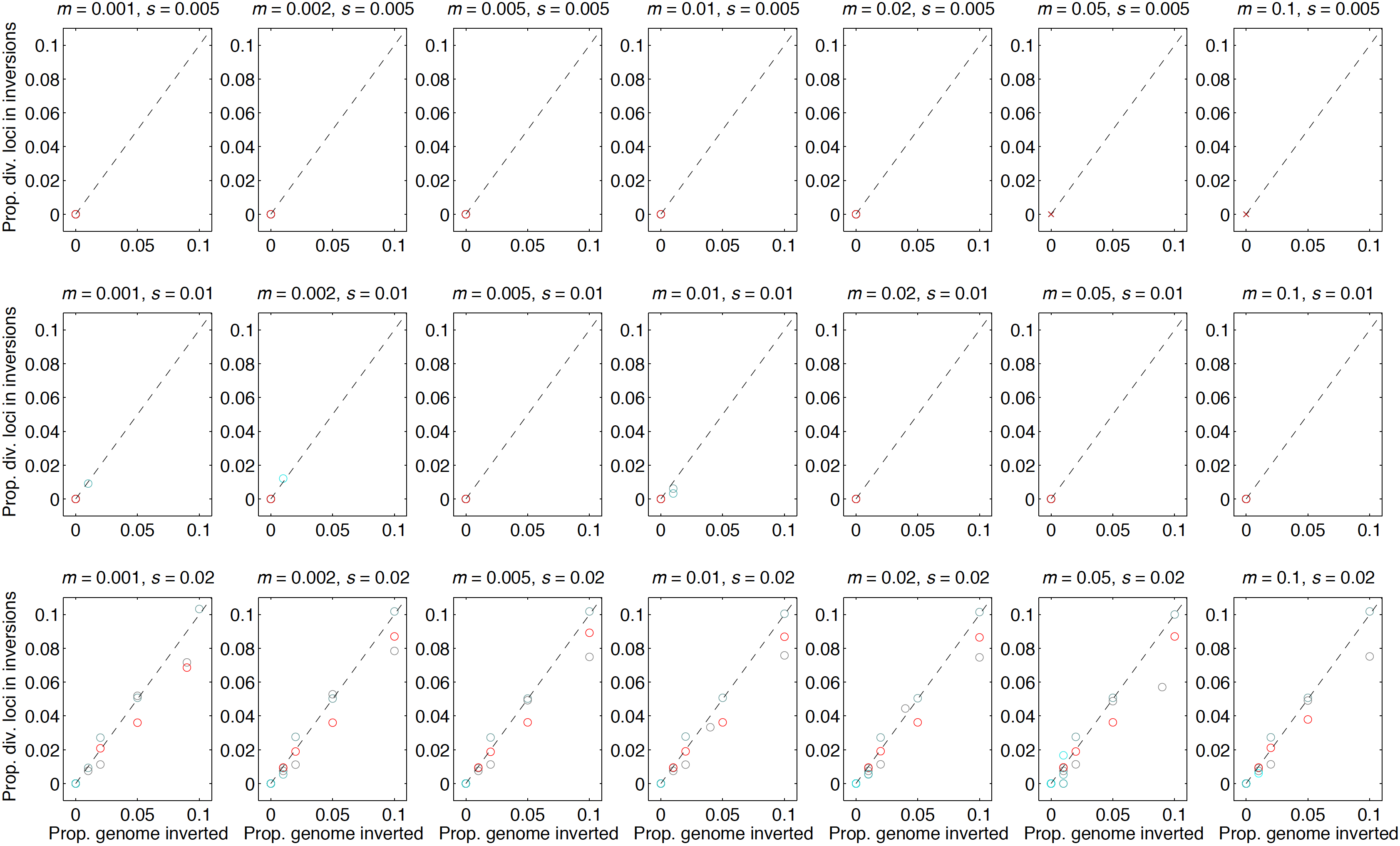


**Figure S1. With smaller inversions starting at a low frequency (*p* = 0.02), there was extremely rare evidence of biased accumulation of divergence within inverted regions.** Circles show runs in which divergence reached a designated barrier strength, *m*/*m*e ≥ 500; “x” symbols show runs that did not reach this barrier strength within the allotted time (1,200,000 mutations and generations). Periods of allopatry were varied in steps of 1000 generations. As colors change from cyan to red, the period of allopatry changes, respectively, from zero to as long as 50,000 generations (if red symbols are not visible, it is because the barrier was reached even prior to the end of the allopatric period). Each inversion spanned a 10-cM portion of a chromosome (equal to 1% of the total genome). Each panel shows results from 35 simulation runs: for a given combination of *m* and *s*, five different numbers of inversions were seeded (0, 1, 2, 5, or 10 inversions) and for each of these, seven different durations of allopatry were explored (0, 5000, 10000, 15000, 20000, 25000, or 50000 generations). Overlap of symbols in some cases obscures data points from view. Many points at the origin ([0,0]) in the upper two rows of panels indicate that nearly all inversions were lost completely (due to the combination of selection, migration, and drift) for those parameter sets, and hence the proportion of the genome that ended up being inverted in such cases was zero.


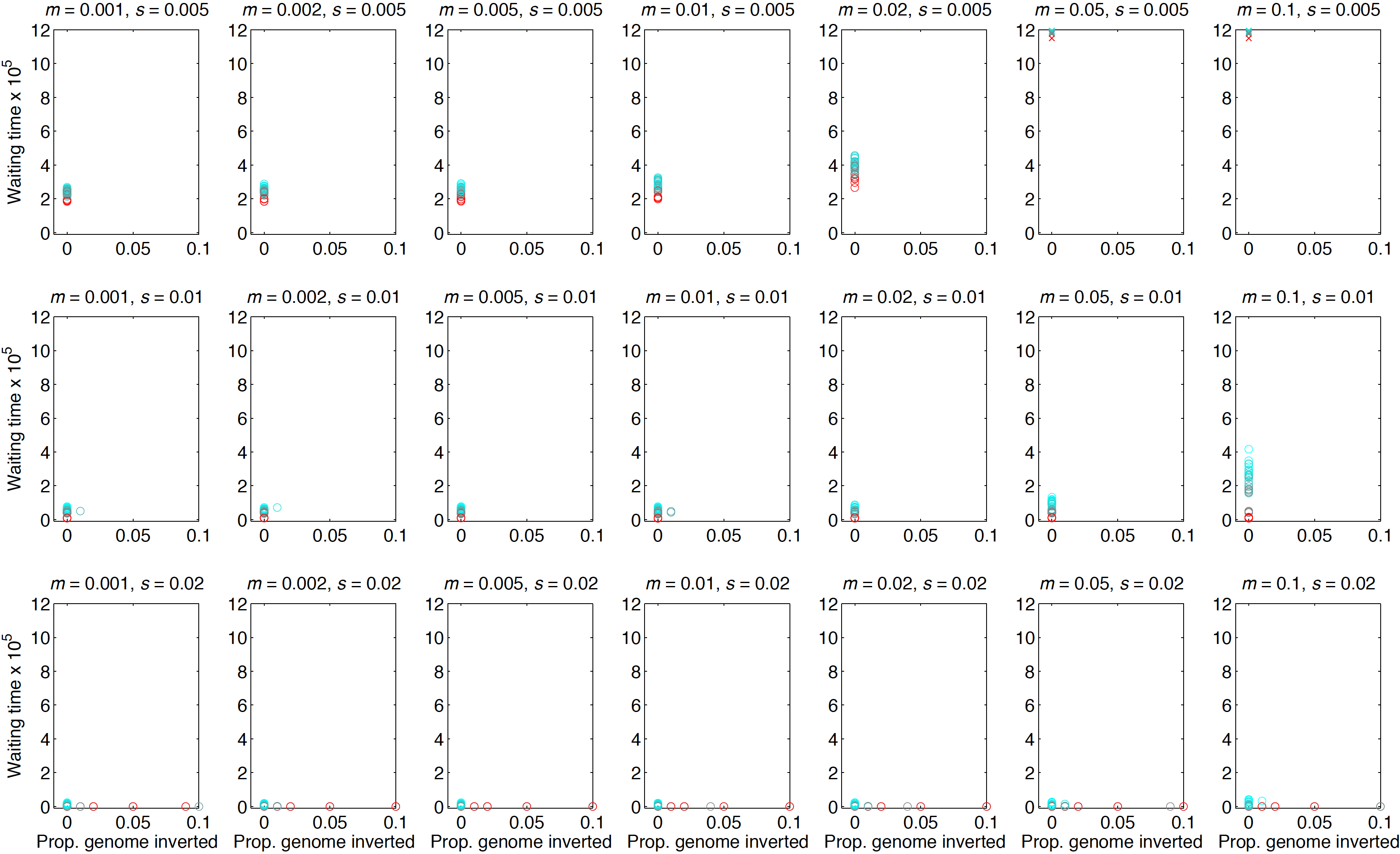


**Figure S2. Waiting times to the designated barrier strength, *b* ≥ 500, for the same set of runs as shown in Figure S1.** Points and panels have the same interpretation as Figure 4 in the main text. However, unlike Figure 4, here there is very rare evidence that inversions sped up speciation. Many panels show no results for a proportion of the genome inverted > 0 because all inversions were lost from the population prior to the ends of the runs in those panels.


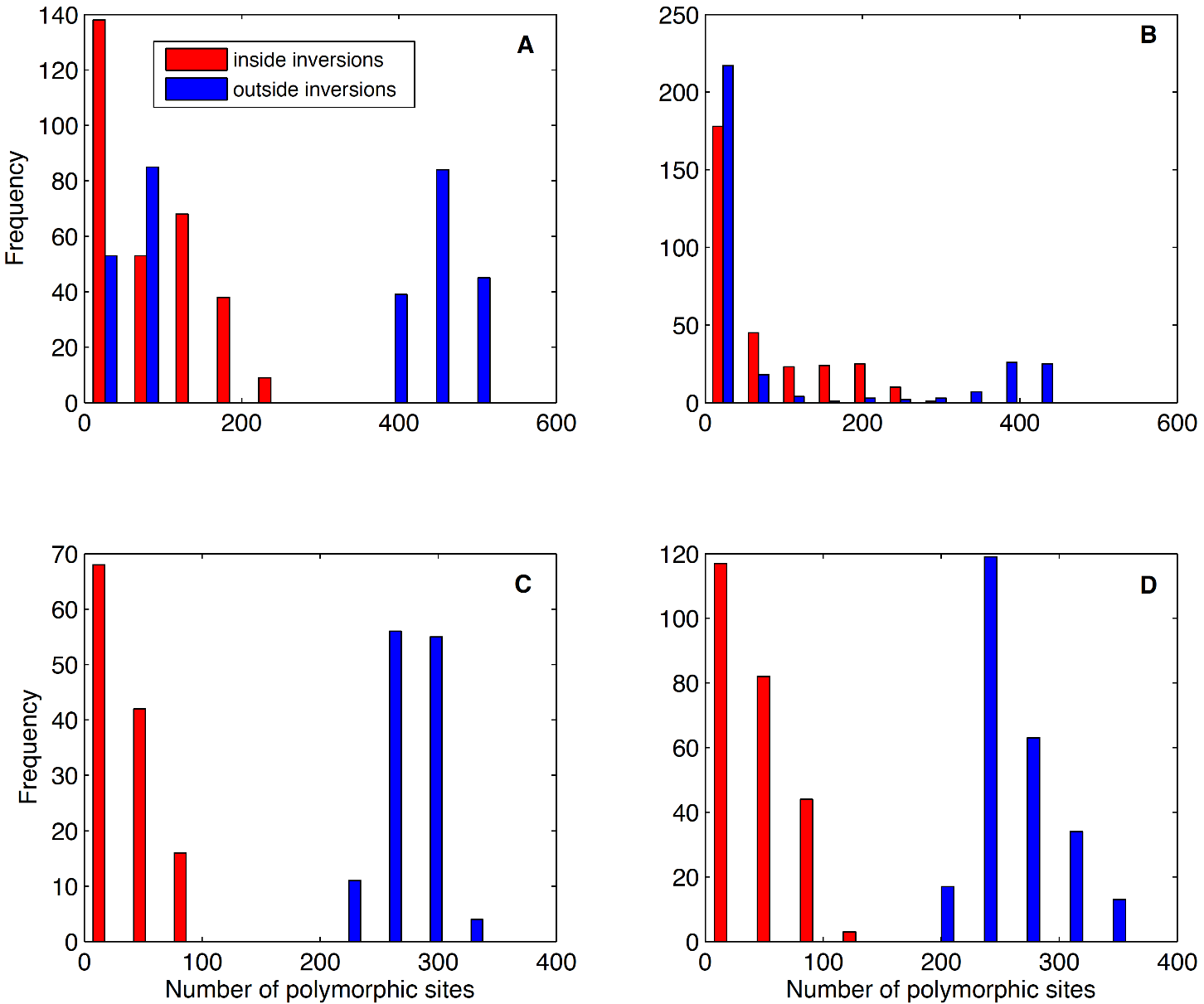


**Figure S3**. The number of polymorphic sites at the end of the runs shown in Figure 3 in the main text. Each panel here corresponds to the panel of the same letter in Figure 3.


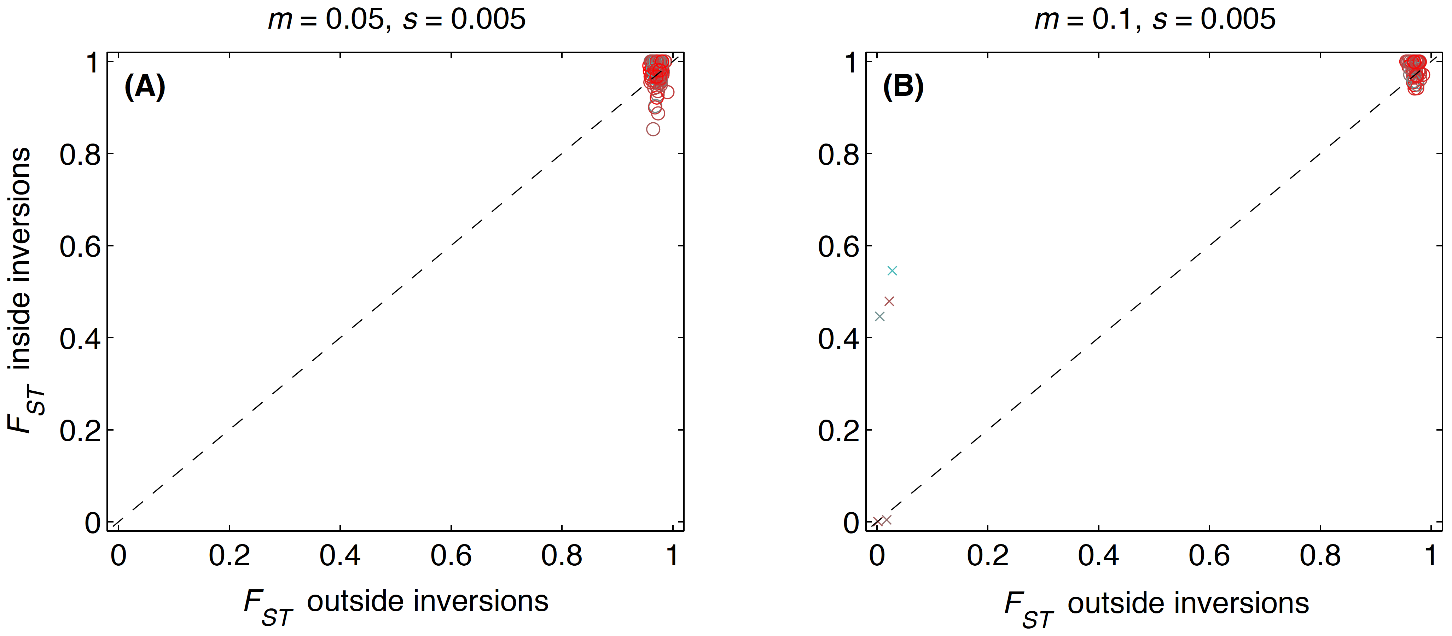


**Figure S4. Average *FST* for loci within vs. outside of inverted regions at the end of simulation runs with a low frequency of inversions (*p* = 0.02) upon secondary contact**. Parameters are the same here as in Figures 2A,B in the main text, with the exception that in Figure 2, inversions were seeded as fixed differences between demes, whereas here inversions started at low frequency in one deme (*p* = 0.02). Interpretation of points is the same as in Figure 2 in the main text. Results are from a total of 1632 simulation runs (816 per panel).


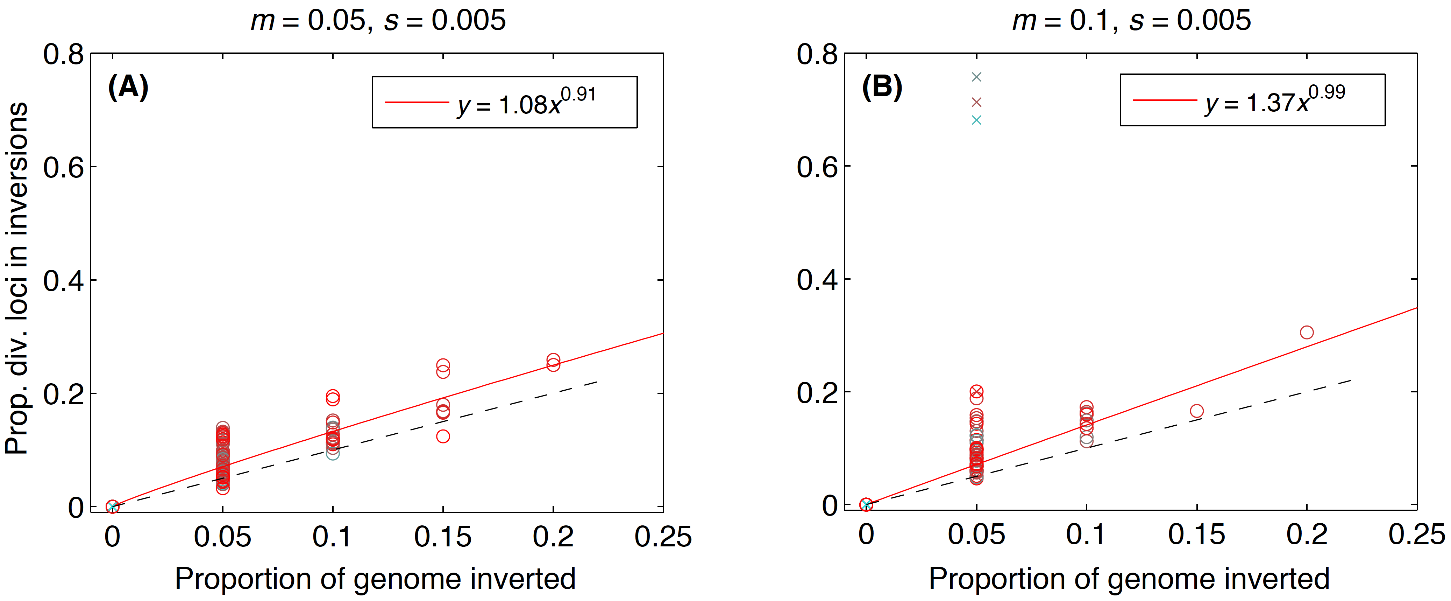


**Figure S5. Biased accumulation of divergence within inverted regions when inversions are seeded at a low frequency (*p* = 0.02) upon secondary contact** (results from the same set of simulation runs as in Figure S4). Parameters are the same here as in Figures 3A,B in the main text, with the exception that in Figure 3 the inversions were seeded as fixed differences. Note that the two parameter combinations shown here are those that were the most favorable for having inversions affect the dynamics of speciation. There are two main points here. (i) As in the main text, divergence accumulated in a biased manner inside inversions, but (ii) there were many fewer opportunities for this to happen because so many of the inversions were lost: though equal numbers of simulations started with 0, 1, 2, 3, 4, and 5 inversions each, in (A) 90% of the inversions were lost, and 77% of those runs starting with one or more inversions had zero at the end; in (B) 93% of the inversions were lost, and 82% of those runs starting with one or more inversions had zero at the end. Hence, even when several, large (50 cM) inversions are present upon secondary contact, they may only affect speciation dynamics a minority of the time if their population frequencies are low. Interpretation of panels and symbols within is the same as for Figure 3 in the main text. The dashed black line is the null expectation, and the red line is the fit from power law regression through the open circles (runs that reached the designated barrier strength, *m*/*me* = 500, prior to 1.2 million generations elapsing), forced through the origin.


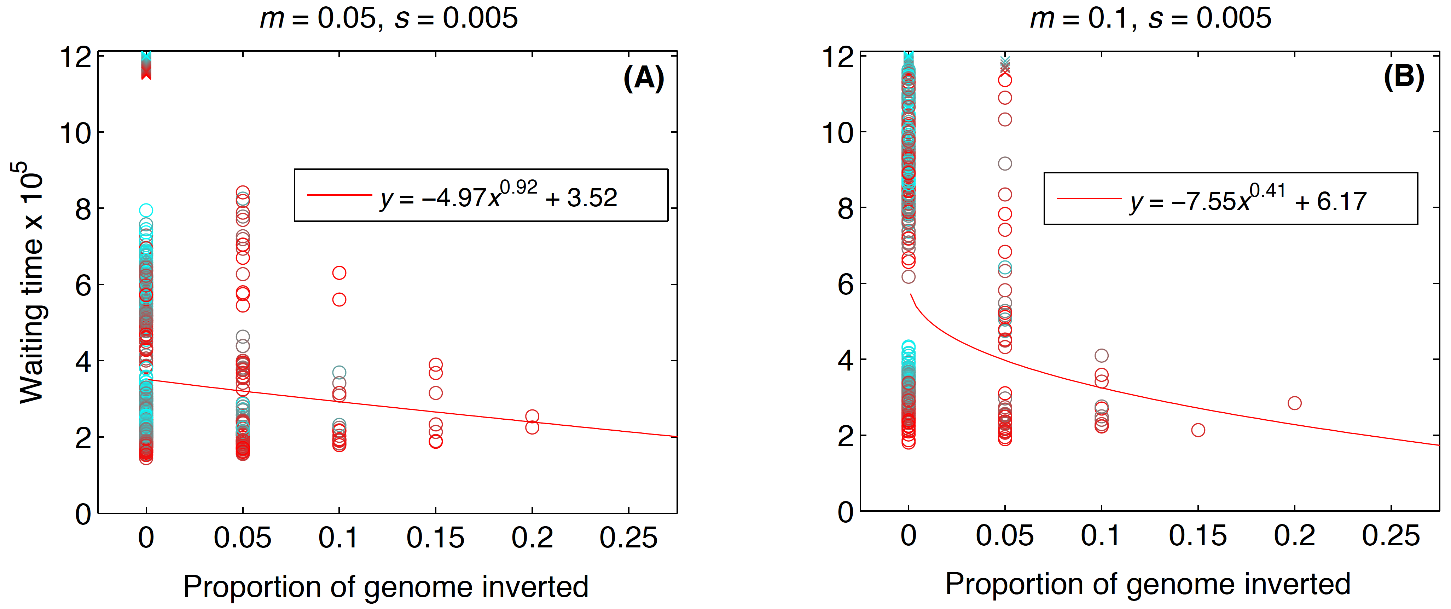


**Figure S6. Waiting times to the designated barrier strength (*m*/*me* = 500) when inversions are seeded at a low frequency (*p* = 0.02) upon secondary contact**. Results are for the same set of simulation runs as shown above in Figures S4 and S5. Parameters are the same here as in Figures 4A,B in the main text, with the exception that in Figure 4 the inversions were seeded as fixed differences. Note that the two parameter combinations shown here are the ones that were the most favorable for having inversions affect the dynamics of speciation. As in Figure S5, there are two main points here. (i) As in the main text, inversions could shorten the waiting times to speciation, but (ii) there were many fewer opportunities for this to happen because so many of the inversions were lost (see legend of Figure S5 for specific numbers). Interpretation of panels and symbols within is the same as for Figure 4 in the main text. The red line is the fit from power law regression through the open circles (runs that reached the designated barrier strength, *m*/*me* = 500, prior to 1.2 million generations elapsing).


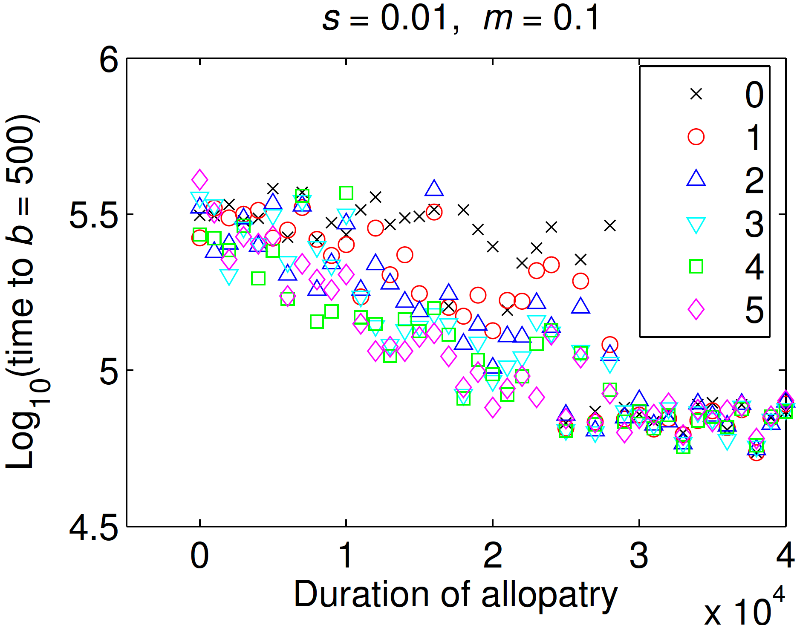


**Figure S7. Examples showing how a pronounced role for inversions in shortening wait times to reach a given barrier strength is dependent upon the duration of allopatry**. Replicate simulations with the same sequences of divergently selected mutations arising were conducted with varying numbers of inversions (0 – 5, legend) seeded as fixed differences (*p* = 1), and with secondary contact beginning at various times (x-axis). Black “x” symbols show results without inversions; other symbols show results with the number of inversions indicated by the legend. Strong elevation of the “x” symbols above the others from about 12,000 – 28,000 generations indicates that, with the parameters used, if allopatry lasted for approximately that duration, having one or more inversions upon secondary contact could substantially shorten the wait time to reach the designated barrier strength (*b* = *m*/*m*e = 500). Note that the y-axis is logarithmically scaled. When allopatry was too short, inversions were unlikely to have captured enough divergently selected loci to help speed the process, because there was not enough time for enough divergently selected mutations to have arisen. When allopatry was too long, enough divergence accumulated prior to secondary contact such that divergence had already reached the “genomic” phase and inversions were thus incidental to the dynamics of the speciation process.
